# Gene relevance based on multiple evidences in complex networks

**DOI:** 10.1093/bioinformatics/btz652

**Published:** 2019-08-22

**Authors:** Noemi Di Nanni, Matteo Gnocchi, Marco Moscatelli, Luciano Milanesi, Ettore Mosca

**Affiliations:** Department of Biomedical Sciences, Institute of Biomedical Technologies, National Research Council, 20090 Segrate (MI), Italy; Department of Industrial and Information Engineering, University of Pavia, Italy; Department of Biomedical Sciences, Institute of Biomedical Technologies, National Research Council, 20090 Segrate (MI), Italy; Department of Biomedical Sciences, Institute of Biomedical Technologies, National Research Council, 20090 Segrate (MI), Italy; Department of Biomedical Sciences, Institute of Biomedical Technologies, National Research Council, 20090 Segrate (MI), Italy; Department of Biomedical Sciences, Institute of Biomedical Technologies, National Research Council, 20090 Segrate (MI), Italy

## Abstract

**Motivation:**

Multi-omics approaches offer the opportunity to reconstruct a more complete picture of the molecular events associated with human diseases, but pose challenges in data analysis. Network-based methods for the analysis of multi-omics leverage the complex web of macromolecular interactions occurring within cells to extract significant patterns of molecular alterations. Existing network-based approaches typically address specific combinations of omics and are limited in terms of the number of layers that can be jointly analysed. In this study, we investigate the application of network diffusion to quantify gene relevance on the basis of multiple evidences (layers).

**Results:**

We introduce a gene score (mND) that quantifies the relevance of a gene in a biological process taking into account the network proximity of the gene and its first neighbours to other altered genes. We show that mND has a better performance over existing methods in finding altered genes in network proximity in one or more layers. We also report good performances in recovering known cancer genes. The pipeline described in this article is broadly applicable, because it can handle different types of inputs: in addition to multi-omics datasets, datasets that are stratified in many classes (e.g., cell clusters emerging from single cell analyses) or a combination of the two scenarios.

**Availability and implementation:**

The R package ‘mND’ is available at URL: https://www.itb.cnr.it/mnd.

**Supplementary information:**

Supplementary data are available at *Bioinformatics* online.

## 1 Introduction

Current omics technologies are capable of generating data relative to different types of molecular entities (DNA, RNA, proteins, etc.). The resulting heterogeneous datasets can be analysed to reconstruct a more complete picture of the molecular events underlying human diseases. The analysis of such datasets is a challenging problem in bioinformatics, due to differences in terms of information type, coverage, data distribution type, noise, just to mention a few and, last but not least, research questions that can be addressed (Ahmad and Fröhlich, 2016; Huang *et al.*, 2017; Ritchie *et al.*, 2015).

Knowledge about the complex web of direct and indirect interactions among macromolecules at genome scale is a powerful resource for explaining multiple omics measurements, highlighting the molecular mechanisms underlying diseases (Kristensen *et al.*, 2014). Indeed, the emergence of a disease can be explained as a combinatorial problem in which different molecular alterations affect a series of pathways that result in a similar phenotype (Barabasi *et al.*, 2011).

In this view, network-based methods exploit known interactions in finding meaningful patterns in omics datasets—such as coherent variations of several functionally related genes (Bersanelli *et al.*, 2016a,b)—and help explain the heterogeneity of alterations detected at gene-level, as the intra-tumour genetic heterogeneity, in terms of interacting genes (e.g. disease modules (Barabasi *et al.*, 2011)). In this last decade, the principle of network diffusion (ND)—also referred to as network propagation—has been proposed to solve several problems in biological data analysis, thanks to its ability to quantify network proximity considering simultaneously all the possible network paths between query network nodes (e.g. genes) (Cowen *et al.*, 2017).

Several studies focused on the integrative analysis of multiple omics datasets using ND. The method TieDIE (Paull *et al.*, 2013) applies ND for identifying a subnetwork that links a source gene set carrying genomic alterations to a target set of differentially expressed genes on the same network. There is evidence that ND is useful in predicting ‘silent’ players in cancer (Ruffalo *et al.*, 2015), using different combinations of diffusion scores (e.g. dot product, Spearman correlation) obtained from two types of initial statistics (mutation frequency and differential expression). More recently, NetICS (Dimitrakopoulos *et al.*, 2018), which uses ND on directed graphs, has been proposed to integrate aberration events [somatic mutations (SMs), copy number variations, methylation and miRNA expression data] with differential expressions to prioritize cancer genes. In general, existing methods are usually relevant only to two types of omics or specific combinations of them.

**Fig. 1.**
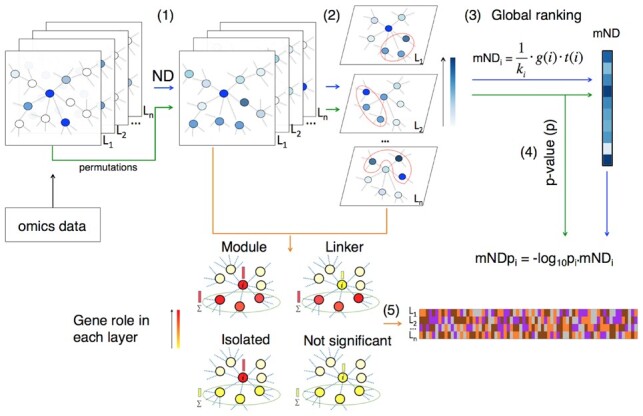
Flowchart of the analysis pipeline with mND. (1) Network-diffusion is applied to the original dataset, composed of multiple layers *L*_1_, *L*_2_, …, *L*_*n*_ (e.g. different types of omics or multiple samples of same omic type); (2) identification of the top *k* neighbours for each gene in each layer; (3) calculation of mND score; (4) empirical *P*-value assessment; (5) classification of genes across layers

In this article, we present a gene-score, named ‘mND’, to assess gene relevance on the basis of gene position in a genome-scale network in relation to one or more types of biological evidences (‘layers’ hereafter) (Fig. 1). Genes are ranked considering their relevance within each layer (e.g. number of mutations, *P*-values from differential expression analysis), their network proximity to other relevant genes as well as the layer-specific relevance of their neighbours. Statistical significance of the gene scores defined by mND (mND scores) is assessed by dataset permutations. To help unravel the role of a gene in each layer, in addition to producing a global gene ranking, mND classifies each gene as a member of a module of high scoring genes, linker of high scoring genes or, lastly, high scoring but isolated gene. Unlike current methods, mND can be used in integrative analysis of different types of omics (e.g. mutation, CNV and expression changes) or multiple samples of same omic type (e.g. patient-level mutational analysis), without particular constraints on the number of layers and layer type. We show that, taking into account ND scores of neighbouring genes, mND has a better ability in finding high scoring genes in network proximity over multiple layers. Furthermore, we account for good performances in recovering known cancer genes in four cancer types, using two types of omics and a single type of omics at patient-level. We show that the application of mND to rank genes based on mutations and expression changes in breast cancer points to relevant pathways underlying the disease, providing a more complete picture than each individual omics on its own. Lastly, layer-specific gene classification suggests functional roles and offers mechanistic insights in relation to the datasets studied.

## 2 Materials and methods

### 2.1 mND

The calculation of mND score requires an undirected interaction network *G* and a matrix of initial scores $X=\left[x_{1},x_{2},\ldots ,x_{L}\right]$, in which each column (representing a layer) is a score vector $x_{i}$ over all vertices of *G*.

The computation of mND consists of five steps (Fig. 1).

*Network diffusion*. Input scores (X) are smoothed by ND, obtaining the corresponding network-constrained scores $X^{*}$, using the following iterative procedure, where the subscript $q\in \left[0,\infty \right)$ indicates the current iteration and $X_{0}=X:$

(1) $$X_{q+1}=\alpha WX_{q}+\left(1-\alpha \right)X_{0}\;\;\;\;\;\;\;\;X^{\mathrm{ss}}=\underset{q\to \infty }{\lim}X_{q}$$

where α∈(0, 1) is a scalar that weights the relative importance of topology and input scores, and ***W*** is the symmetric normalized form of the adjacency matrix ***A***:

(2) $$w_{\mathrm{ij}}=\frac{a_{\mathrm{ij}}}{\sqrt{d_{i}}\cdot \sqrt{d_{j}}}$$

where $a_{\mathrm{ij}}\in A$ are the elements of the adjacency matrix, ${(d}_{i},d_{j})$ are the degrees of the corresponding genes. The final matrix ***X*^ss^** is the matrix $X_{q+1}$ that satisfies the termination criterion $\max\left({|X}_{q+1}-X_{q}|\right)<10^{-6}$. Parameter *α* was set to 0.7, a value that represents a good trade-off between diffusion rate and computational cost, and determined consistent results in previous studies (Bersanelli *et al.*, 2016a, b; Hofree *et al.*, 2013; Mosca *et al.*, 2014, 2017; Vanunu *et al.*, 2010). We estimated the sensitivity of mND to *α* and found that varying *α* by ±10% resulted in highly correlated mND scores and only a few different genes (6–8%) among the top 100 (Supplementary Fig. S1 and Table S1). To enable direct multiplication of values belonging to different layers, ***X*^ss^** is column-wise normalized by the maximum of each column, obtaining the matrix $X^{*}$.

*Neighbours selection.* For each gene *i*, the top $k_{i}=\text{min}(k,d_{i})$ first neighbours with the highest diffusion scores in each layer *l* are selected as representatives of the network proximity of the neighbourhood of *i* to the original scores in layer *l*, and their ND scores are summed:

(3) $$T\left(i,l\right)=\text{max}\left\{\left.\sum_{j\in C}a_{\mathrm{ij}}x_{\mathrm{jl}}^{*}\;\right|C\in S\right\}$$

where$\;x_{\mathrm{jl}}^{*}\in X^{*}$ with $j=\left\{1,\;2,\;\ldots ,N\right\}$ is the network-constrained value of *j-th* gene in *l-th* layer (j≠i), S is the set of all $k_{i}$-subsets of $\left\{1,\;2,\;\ldots ,N\right\}$, and $0<T\leq k_{i}$.

We explored the performance of mND at varying *k* and found *k* = 3 to be a reasonable choice (see Section 3). Further, we evaluated the sensitivity of mND to the value of *k* and found that varying *k* of one unit had only minor effects on mND scores, which are highly correlated and indeed differ of only a few (∼4–6) genes among the top 100 (Supplementary Fig. S2 and Table S2). An opportunity to further optimize the value of *k* relies in selecting a value that yields connected networks enriched in initial scores (Supplementary Methods).

*Integration.* At this point, the mND score for gene *i* is calculated as the product between the sum of its network constrained scores (term $g\left(i\right)$) and the sum of the contributions of its top *k* first neighbours (term $t\left(i\right)$):

(4) $${\mathrm{mND}}_{i}=\frac{1}{k_{i}}\;g\left(i\right)\;t\left(i\right)=\;\frac{1}{k_{i}}\left(\sum_{l=1}^{L}x_{\mathrm{il}}^{*}\right)\left(\sum_{l=1}^{L}T\left(i,l\right)\right)$$

where *L* is the total number of layers and $0<{\mathrm{mND}}_{i}\leq L^{2}$.

*Significance assessment*. The corresponding values of ${\text{mND}}_{i}^{†}$, obtained with permuted versions of X, are used to calculate empirical *P-*values, i.e. the fraction of times ${\text{mND}}_{i}^{†}\;\geq {\text{mND}}_{i}$. The product of $p_{i}$ and ${\text{mND}}_{i}$

(5) $${{\text{mNDp}}_{i}}_{\;}=-{\text{log}}_{10}\left(p_{i}\right)\cdot {\text{mND}}_{i}$$

provides a gene score weighted by its estimated statistical significance, as previously described (Bersanelli *et al.*, 2016b; Xiao *et al.*, 2014).

*Classification.* Lastly, a gene *i* is classified by evaluating the membership of the gene in two gene sets *H_l_* and *N_l_* which define, respectively, the high scoring genes according to original data (X) and neighbour information (T). The gene set *H_l_* is composed of the high scoring genes in layer *l* of X, defined using a layer-specific criterion (e.g. the differentially expressed genes at *P *<* *0.05). The gene set *N_l_* is composed of the genes with the highest

(6) $${\text{tp}}_{il}=-{\text{log}}_{10}\left({p_{\mathrm{il}}^{t}}_{\;}\right)\cdot T\left(i,l\right)$$

where ${p_{\mathrm{il}}^{t}}_{\;}$ is the empirical *P*-value calculated comparing T to T^†^, the latter obtained with permuted ***X***. The use of empirical *P*-value to scale T overcomes the issue of ties due to genes with equal values of T. The cardinality of *N*_*l*_ can be defined several ways: considering an *ad hoc* number of top values (e.g. in proportion to |*H*_*l*_|), on the basis of ${p_{\mathrm{il}}^{t}}_{\;}$or a combination of the two criteria. The gene *i* is ISOLATED if it is in *H*_*l*_ but its neighbourhood is not in *N_l_.* If both the gene and its neighbourhood are in, respectively, *H*_*l*_ and *N*_*l*_ the gene is part of a high scoring module and therefore termed MODULE. If the gene is not in *H*_*l*_ but its neighbourhood is in *N*_*l*_, then it is named as LINKER.

The computational cost of mND depends on interactome size (number of nodes and links), number of layers and number of permutations used in significant assessment. In particular, ND is the rate-limiting step, which is repeated several times during significance assessment. For example, the computation of ND using STRING (11 796 genes and 309 850 links) on two layers of initial scores required approximately 30 s on a server with dual Intel(R) Xeon(R) CPU E5-2697 v3 @ 2.60 GHz, 64GB DDR4 2133 MHz memory and disk storage on Lustre Filesystem; the whole analysis, involving 1000 permutations, took about 1 h and 50 min on 4 cores. See Supplementary Table S3 for additional details and further examples.

### 2.2 Macromolecular interactions

Three sources of interactions were considered, abbreviated as STRING (11 796 genes; 309 850 interactions) (Szklarczyk *et al.*, 2015), GH (13 244; 138 045) (Ghiassian *et al.*, 2015) and WU (6016; 128 150) (Wu *et al.*, 2010). Native identifiers were mapped to Entrez Gene (Brown *et al.*, 2015) identifiers using the R package ‘org.Hs.eg.db’ (Carlson, 2018).

### 2.3 Analysis of somatic mutations and gene expression variations

SMs and gene expression (GE) data from matched tumour-normal samples (blood for SM and solid tissue for GE) were collected from The Cancer Genome Atlas (TCGA) (Tomczak *et al.*, 2015) for breast invasive carcinoma (BC), lung squamous cell carcinoma (LUSC), prostate adenocarcinoma (PRAD) and thyroid carcinoma (THCA), using the R packages TCGAbiolinks (Colaprico *et al.*, 2016) and isma (Di Nanni *et al.*, 2019) and considering the human genome version 38 (hg38).

Mutation Annotation Format files were obtained from four pipelines: Muse (Fan *et al.*, 2016), Mutect2 (Cibulskis *et al.*, 2013), SomaticSniper (Larson *et al.*, 2012) and Varscan2 (Koboldt *et al.*, 2012). Only mutation sites detected by at least two variant callers were considered. Gene mutation frequencies were calculated as the fraction of subjects in which a gene was associated with at least one mutation. Gene expression data were obtained using the TCGA workflow ‘HTSeq-Counts’. The R package limma (Ritchie *et al.*, 2015) was used to normalize and quantify differential expression in matched tumour-normal samples, yielding log-fold changes, the corresponding *P*-values and FDRs (BH method).

The four cancer datasets were considered in two tasks: the analysis of two types of omics, mutations and expression changes, and the analysis of mutation profiles of multiple patients. In the first task, $x_{1}$ was defined as gene mutation frequencies while $x_{2}$ as –log_10_ (FDR). In the second task, each layer $x_{i}$ was represented by mutation profiles of subjects, defined as the number of mutation sites in each gene. In all analysis, empirical *P*-values were calculated on a total of 1000 permutations (the input matrix and 999 random permutations of it).

In the joint analysis of mutations and expression changes in BC, the two sets of high scoring genes (*H*_1_, *H*_2_) were defined considering, respectively, all genes with at least one mutation (1238 genes) and the top 1200 differentially expressed genes (FDR < 10^−7^). We observed that *k *=* *3 was a reasonable choice to obtain connected gene networks enriched in genes with the highest mutation frequencies and expression variations (Supplementary Methods and Fig. S3).

### 2.4 Signal assignment to gene modules and performance assessment in finding significant genes that lie in network proximity

Each gene module was defined as the largest connected component obtained considering the genes associated with a biological pathway (from KEGG database (Kanehisa *et al.*, 2017)) and all interactions among them in GH interactome (Supplementary Fig. S4). The highest and lowest values of gene mutation frequencies (***x*_1_**) and fold changes (***x*_2_**) calculated from BC data (see above) were used to define, respectively, high scoring genes and low scoring genes (Supplementary Fig. S5). High scoring values were randomly assigned to genes of each module independently for $x_{1}$ and $x_{2}$, in thus to obtain a specific percentage (e.g. 10%) of high scoring genes within the module in each layer. Unused high scoring values were assigned to genes outside the module and, lastly, low scoring values were assigned to the remaining genes within and outside the module. Recall was defined as the fraction of module genes ranked (by the assessed method) among the top *M* genes, where *M* is the module size. Recall was assessed using, beyond mND score, the product of ND scores (‘NDPROD’) between the two layers (as in Ruffalo *et al.* (2015)), the minimum of ND scores (‘NDMIN’) between the two layers (as in TieDIE, Paull *et al.*, 2013) and the rank product (‘RP’) of initial scores.

### 2.5 Evaluating performance in recovering known cancer genes

The partial area under the ROC curve (pAUC) was used to quantify the performance of methods in recovering known cancer genes at low false positive rates. This measure accounts for the number of true positives that score higher than the *n*-th highest scoring negative, measured for all value from 1 to *n*:

(7) $${\text{pAUC}}_{n}=\frac{1}{n\mathrm{TP}}\sum_{i=1}^{n}{\text{TP}}_{i}$$

where TP is the total number of known cancer genes and TP_*i*_ is the number of true positives that score higher than the *i-*th highest scoring negative (Scott and Burton, 2007). We calculated pAUC_*n*_ to evaluate which method had low false positive rates in prioritizing genes whose mutation or differential expression was associated with the considered cancer. Genes mutations associated with cancer were collected from COSMIC (Tate *et al.*, 2019) and previous studies (Kandoth *et al.*, 2013; in Lawrence *et al.*, 2014). Differentially expressed genes were derived from Bioexpress (Dingerdissen *et al.*, 2018), considering log_2_-fold change between matched primary tumour-normal samples greater than or equal to 1 and FDR < 0.05. NetICS was downloaded from https://github.com/cbg-ethz/netics.

### 2.6 Pathway analysis

Pathways were downloaded from the KEGG database (Kanehisa *et al.*, 2017). A total of 331 human pathways with at least five genes were considered. The number of genes prioritized in each pathway by mND, by gene expression ($x_{2}^{\;})$, ND scores of gene mutation frequencies ($x_{1}^{*}$) and gene expression ($x_{2}^{*})$ (Supplementary Table S4A–D, respectively), were quantified for different numbers of top ranking genes (*n* ={50, 100, 150, 250, 300}). For each pathway and value of *n*, the difference DP(*n*) between the number of genes (*D*) found by mND and the best of the other approaches was quantified as (Supplementary Table S4E):

(8) $$\text{DP}(n)=D_{\text{mND}}(n)-\max\left(D_{x_{2}^{}}(n),\;D_{x_{1}^{*}}(n),\;D_{x_{2}^{*}}(n)\right)$$

## 3 Results

Following is the presentation of the performance of mND in the general problem of locating significant genes that lie in network proximity, using random permutations of real omics data on gene modules representing biological pathways. The assessment of its ability in recovering known cancer genes, a problem considered by recent network-based multi-omics methods (Dimitrakopoulos *et al.*, 2018; Paull *et al.*, 2013; Ruffalo *et al.*, 2015). Lastly, the description of the results obtained applying mND on gene mutations and GE changes observed in BC.

### 3.1 Finding significant genes that lie in network proximity

**Fig. 2.**
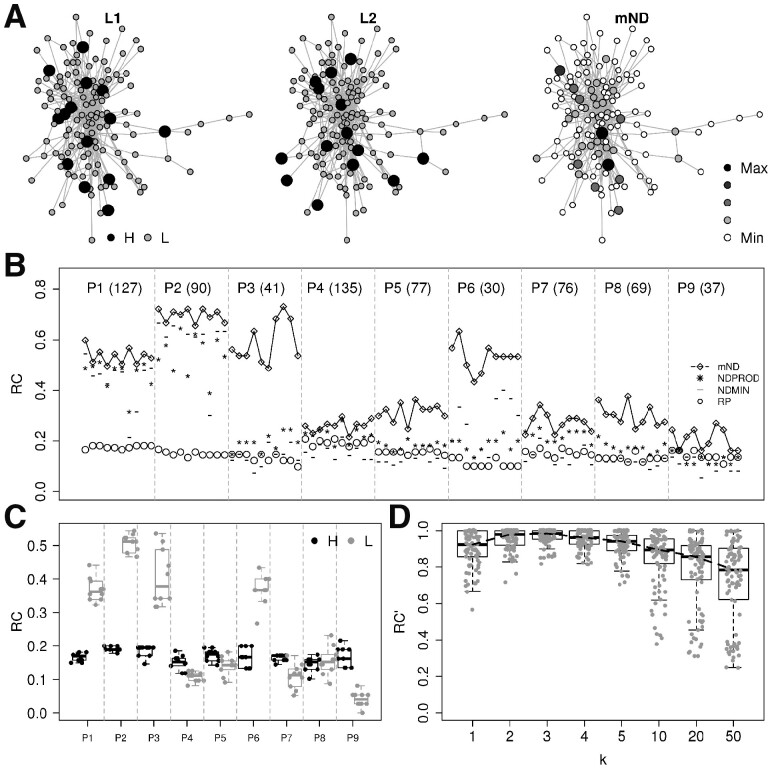
Performance in ranking high scoring genes in network proximity. (**A**) Example of a gene module with its high scoring genes (H, black) in each of the two layers and the resulting mND score; only genes belonging to the module and links occurring among such genes are reported. (**B**) Recall values for 10 signal permutations for each of the nine modules (P1, P2, …, P9), using mND score and other methods; the number between parentheses after module id is module size. (**C**) Recall values, shown separately for high scoring genes and other genes in each module. (**D**) Recall values normalized by the highest recall found for each input configuration at varying number of neighbours (*k*). (A–D) These results were obtained using interactome GH

To assess the ability of mND in finding high scoring genes in network proximity across multiple layers, we assigned two types of real signal (gene mutation frequencies and log-fold changes) to gene modules of different size and modularity, corresponding to real pathways (Fig. 2A, Supplementary Figs S4 and S5).

In each of the resulting configurations, we compared the recall values (see Materials and methods) obtained by mND to those obtained by other methods. The rank product (RP) was successful in identifying genes with high scoring values in at least one of the two layers (Fig. 2B), but typically missed other module genes with lower values. NDPROD, a multi-omic approach described in Ruffalo *et al.* (2015) and corresponding to using only the term g(i) in Equation (4), led to better performance than RP in more than half of the cases, and equal or even low performance in others, indicating the failure to identify high scoring genes in favour of genes in network proximity to the module, but outside of it (Fig. 2B). Similarly, NDMIN, the multilayer combination strategy underlying TieDIE method (Paull *et al.*, 2013), yielded recall values that are higher or lower than RP depending on gene module and signal distribution. Instead, mND determined the highest recall in almost all cases. This result underlines the importance of using gene neighbourhoods, i.e. the term t(i) in Equation (4) (Fig. 2B). Importantly, the performance of mND is the result of spotting both high scoring genes (almost all) plus other module genes with low score, but relevant topological position (Fig. 2C).

Overall, a small number of neighbours (parameter *k* in Equation (4)) was sufficient to guarantee the highest performances (Fig. 2D), which were observed around *k *=* *3. We observed a similar trend when finding significant genes lying in network proximity over three layers (Supplementary Fig. S6).

To assess whether the results obtained in ranking high scoring genes lying in network proximity (Fig. 2) were limited to the interactome in use (GH), we repeated the same analyses using a different interactome (STRING). We observed the same patterns in terms of mND performance, types of genes found and the relation between performance and *k* parameter (Supplementary Fig. S7).

### 3.2 Recovering known cancer genes

**Fig. 3.**
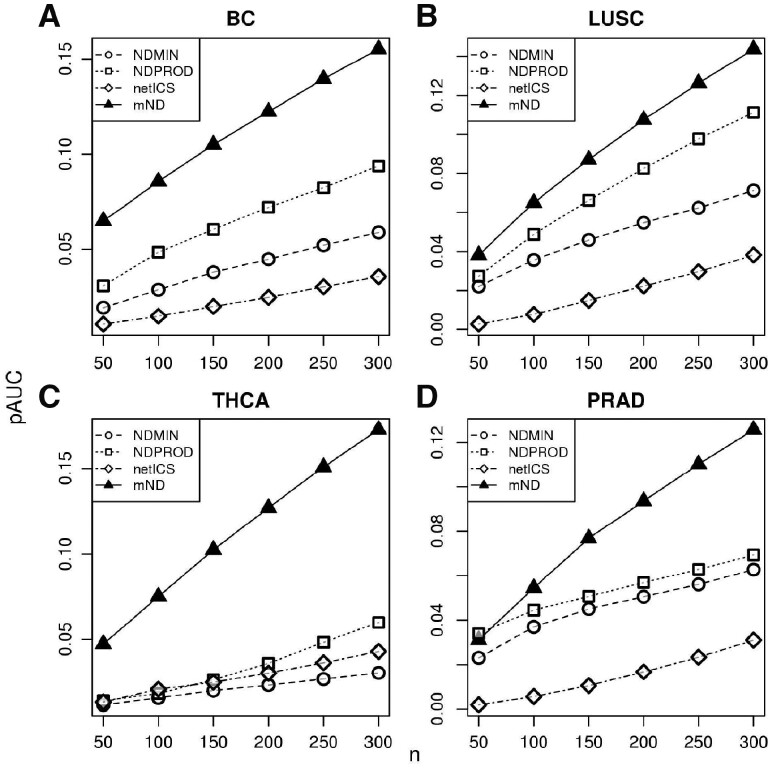
Performance in recovering known cancer genes. Partial AUC (pAUC) at varying number of top false positive ranking genes (*n*) in the analysis of mutations and expression changes in four cancer types. (**A**–**D**) These results were generated using interactome WU

We evaluated the performance of mND in the problem of recovering known cancer genes in four cancer types. Considering mutations and expression changes as input, mND reported higher pAUC than other network-based methods in all four cancer types considered (Fig. 3). We also studied the performance using mutational profiles only as input. In this case, mND reported better performance than other methods in three out of four datasets in recovering genes whose mutations are associated with cancer (Supplementary Fig. S8), while it was the best method in using mutation profiles to recover both mutated and differentially expressed genes involved in cancer (Supplementary Fig. S9).

### 3.3 Gene networks enriched in mutations and expression changes in BC

**Fig. 4.**
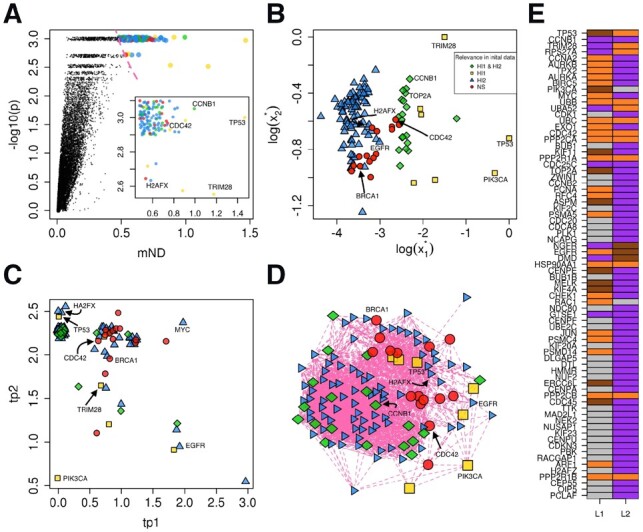
Analysis of mutations and expression changes in BC. (**A**) mND score and empirical *P*-value; the red dashed line indicates the top 123 genes (subplot); colours and shapes have the same meaning of panel B. (**B**) Gene diffusion scores of the top 123 genes ranked by mND. (**C**) tp values (Equation 6) for the two layers. (**D**) Gene network composed of the top 123 genes ranked by mND; colours and shapes have the same meaning as in panel B. (**E**) Classification of genes across layers (only the top 75 ranked genes are shown for clarity); brown: isolated; orange: linker; purple: module; grey: not significant. (A–D) Layer 1 (L_1_): mutations; Layer 2 (L_2_): expression variations. H_1_, H_2_: sets of genes with high initial scores in respectively L_1_ and L_2_. NS: not significant, genes not belonging to H_1_ and H_2_. Green rhombuses: genes belonging to H_1_ and H_2_; blue triangles: genes belonging only to H_1_; yellow rectangles: genes belonging only to H_2_; red shapes: genes neither in H_1_ nor in H_2_. These results were generated using interactome STRING

As a proof of principle, we applied mND to find functionally related genes on the basis of gene mutation frequency (layer 1, L1) and GE variation (layer 2, L2) in BC. Genes highly ranked by mND (Fig. 4A) include those that were relevant according to initial scores in both layers (Fig. 4B, e.g. *CCNB1*, *TOP2A*), as well as those that were high scoring in one of them (Fig. 4B, e.g. *EGFR* and *PIK3CA*), and linker genes (Fig. 4B, red circles), which have low initial values, but lie in relevant network proximity to significantly altered genes. Interestingly, top scoring linker genes include genes already known to be involved in BC, such as *CDC42* and *BRCA1* (Fig. 4B and C). To assess whether genes highly ranked by mND are in significant network proximity, we used network resampling (Bersanelli *et al.*, 2016b): this computational approach calculates a network score considering top ranking genes and shows to which extent such network score is expected if links among genes are shuffled (keeping the same degree distribution). This procedure confirmed that genes highly ranked by mND are in significant network proximity (Supplementary Fig. S10): in particular, a dense module of 123 genes was identified (Fig. 4D).

Gene classification underlined gene roles in each layer, which suggest possible underlying molecular mechanisms (Fig. 4E). For instance, *TP53* is classified as ‘isolated’ according to mutations and ‘linker’ on the basis of GE, because it is highly mutated and its interacting partners are mainly differentially expressed rather than mutated. *CDC42* is classified as linker in both layers: it neither carries a relevant amount of mutations nor is among the top differentially expressed genes, but its interacting partners are highly enriched in both mutations and differential expression. Interestingly, *CDC42* is an important molecule in luminal BC, with prognostic significance (Chrysanthou *et al.*, 2017). Among genes highlighted as modules, we found *PIK3CA* (a highly mutated gene in BC (Mukohara, 2015)), highly ranked on the basis of mutations.

**Fig. 5.**
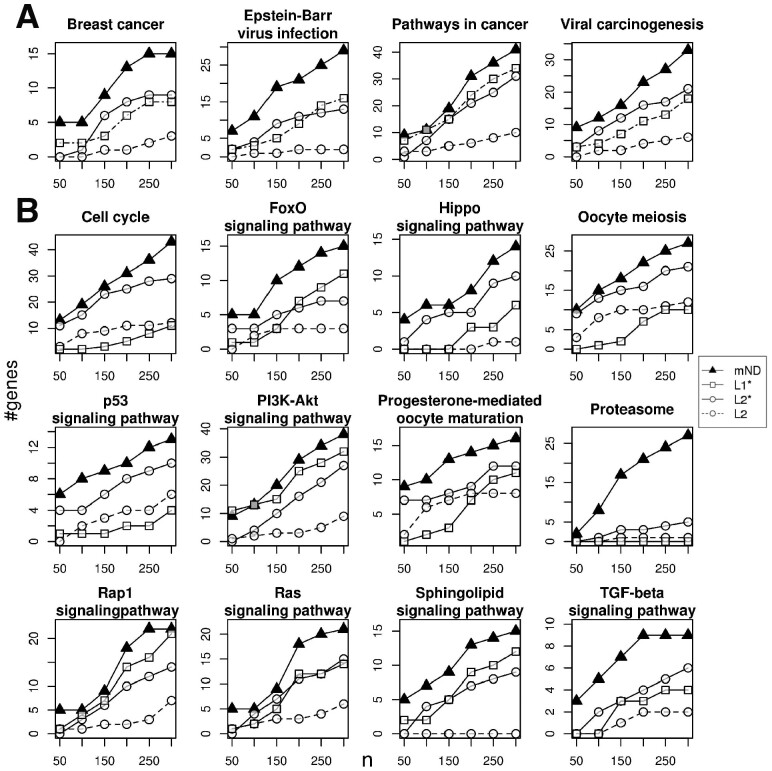
Pathways enriched in mutated genes and/or differentially expressed genes in BC. Number of genes found by mND and single omics analyses (L1*, L2* and L2) in each pathway at varying number of top ranking genes considered (horizontal axis, *n*); L1: mutations; L2: gene expression variations; the asterisk distinguishes between gene ranking by original data and the corresponding network diffusion scores. (**A**) Disease pathways; (**B**) other pathways. (A and B) Pathways from KEGG database

We characterized the genes prioritized by mND in terms of biological pathways. Interestingly, among the pathways in which mND found relatively more genes than each omics considered independently, we found KEGG ‘Breast Cancer’ and signal transduction ways known to have a relevant role in BC (Fig. 5), like ‘Cell Cycle’ (Liu *et al.*, 2008), ‘Hippo signalling pathways’ (Wei *et al.*, 2018), ‘FoxO Signalling pathways’ (Farhan *et al.*, 2017), ‘p53 Signalling pathways’ (Gasco *et al.*, 2002), ‘PI3K-Akt signalling’ (Paplomata and Regan, 2014) and ‘Proteasome’ (Mani and Gelmann, 2005).

## 4 Discussion

Multi-omics analyses, patient-level analyses and multi-classes analyses (e.g. multiple cell clusters) demand methods to highlight the importance of altered genes considering, respectively, different types of summary information across subjects or subject-specific molecular profiles. At the same time, to explain complex patterns in these datasets (e.g. the heterogeneity of mutation profiles of tumour samples) it is important to consider the complex web of macromolecular interactions, which provides known relations among the variables (e.g. genes) under analysis. Recently, the use of first neighbours has been proposed in network-based methods for the analysis of single omics (Gwinner *et al.*, 2017; Horn *et al.*, 2018) and recommended for multi-omics analyses (Modos *et al.*, 2017).

The approach described in this work (mND) highlights genes with a significant network position considering multiple types of biological evidence. Importantly, since mND relies on the mathematical machinery of ND, it prioritizes genes considering their own importance (in proportion to original evidences) and the importance of their network location. ND scores are used to quantify the topological relevance of a gene in the context of the distribution of the considered evidences throughout the entire network and, in particular, mND uses layer-specific highly ‘informative’ first neighbours.

We have shown that mND has a good performance in the general problem of locating significant genes in network proximity using multiple evidences. This problem is involved in several applications in which multi-omics datasets are explained relying on the architecture of intracellular circuits, underlying ‘hot’ gene modules (e.g. disease modules) supported by multiple layers of information.

In the analysis of mutations and differential expression in BC—two types of omics with relevant differences for data analysis in terms of distribution and sparsity—mND prioritized genes carrying both types of alterations, genes associated with one type of alteration and linkers genes, extracting knowledge from both layers. If this should not be the case, a simple solution could be to add the appropriate coefficients in the two sums of Equation (4), in order to weight each layer in relation to the research questions under investigation. The joint analysis of the two omics led to enrichment in relevant pathways, compared to single omics on its own, a result that underlies the added value of combining multiple evidences with mND.

Beyond gene global ranking, mND classifies genes in each layer as members of a module, linkers or isolated genes, on the basis of the amount of signal found in the genes themselves and their neighbours. Complementing the global ranking with layer-by-layer information on gene positions, such classification helps clarifying genes role in the context of the alterations detected. For instance, *TP53* clearly emerges as a gene with primary role in BC, not only because of its mutation, but also because its functional partners are differentially expressed (it is classified as linker in GE layer); *CDC42* is considered important in the molecular mechanisms underlying BC (Chrysanthou *et al.*, 2017), despite being not reported as significantly altered in the considered dataset: indeed, its functional partners include both mutated genes and differentially expressed genes; other genes play a role according to one type of alteration only, like *CDCA8* (Phan *et al.*, 2018), which emerged as being involved specifically in terms of differential expression, being a member of a differential expression module. In the analysis of mutation profiles at single patient level, gene classification underlined the presence of several linkers with a relevant role in BC (Supplementary Fig. S11). For instance, the deletion of *HIC-1*, never found mutated in the dataset under analysis but spotted as linker in 15 subjects, has been demonstrated to promote BC (Cheng *et al.*, 2014; Wang *et al.*, 2018); *FYN* has been proposed as a prognostic marker in ER+ BC (Elias and Ditzel, 2015) and promotes mesenchymal phenotypes of basal types BC cells (Lee *et al.*, 2018).

mND introduces an important advance in the class of multi-omics methods: the applicability of the approach is broad in terms of data types and experimental designs. Indeed, mND works on a general gene-by-sample input matrix, where each column is a vector of scores representing different data types (e.g. genomics, transcriptomics) or the same type (e.g. fold changes or *P*-values from single cell clusters).

Interestingly, we reported good performance also in recovering known cancer genes, a problem addressed by recent network-based methods for the analysis of multi-omics datasets.

In conclusion, the results described in this article support the use of mND for global ranking of genes considering multiple evidences. The results generated by mND can be further processed with other existing tools, for example to characterize the top ranking genes using current annotations (e.g. pathways) or network theory (e.g. centrality measures). At present, mND applies to an interactome with a fixed topology and without edge directions. The generalization of mND pipeline to include layers with different topologies as well as the inclusion of edge directions are interesting opportunities for future developments. However, the latter information is currently lacking for most PPIs and would imply a significant reduction of coverage in terms of the genes studied. As all network-based methods, the performance of mND is bounded by the reliability of current models that describe intracellular circuits. As the data about macromolecular interactions will become more and more available and reliable, network-based analyses will be less affected by the lack of a reference human interactome (Luck *et al.*, 2017). In this context, the impact of tools like mND in molecular biology will presumably increase.

## Supplementary Material

btz652_Supplementary_Data
