# Supplementary material for: Gene relevance based on multiple evidences in complex networks
Source: Bioinformatics. 2019 Aug 22;36(3):865–71. doi: 10.1093/bioinformatics/btz652 (PMC9883679; doi:10.1093/bioinformatics/btz652)
Supplement: btz652_Supplementary_Data [file bioinformatics_36_3_865_s6.zip › SupplementaryFile.docx]

**SUPPLEMENTARY MATERIAL**

**Gene relevance based on multiple evidences in complex networks.**

Noemi Di Nanni^1,2^, Matteo Gnocchi^1^, Marco Moscatelli^1^, Luciano Milanesi^1^, Ettore Mosca^1,*^

^1^Institute of Biomedical Technologies, CNR, Viale Fratelli Cervi 93, 20090, Segrate (MI), Italy, ^2^Department of Industrial and Information Engineering, University of Pavia, Italy

**Supplementary Methods**

- Choice of k evaluating the distribution of initial scores in the gene networks identified by mND

**Supplementary Figures**

- Supplementary Figure 1. Sensitivity of mND to α parameter.

- Supplementary Figure 2. Sensitivity of mND to the value of *k*.

- Supplementary Figure 3. Effect of varying *k* on the enrichment of the top gene networks in high initial scores.

- Supplementary Figure 4. Gene modules.

- Supplementary Figure 5. Initial gene scores assigned to gene modules.

- Supplementary Figure 6. Recall values in the analysis of 3 layers.

- Supplementary Figure 7. Performance in ranking high scoring genes in network proximity in STRING.

- Supplementary Figure 8. Performance in recovering mutated genes associated with cancer.

- Supplementary Figure 9. Performance in recovering known cancer genes.

- Supplementary Figure 10. Network resampling on breast cancer data from TCGA.

- Supplementary Figure 11. Classification of genes across layers in the integration of mutation profiles of subjects.

**Supplementary Tables**

- Supplementary Table 1. Sensitivity of mND to $\alpha$ parameter.

- Supplementary Table 2. Sensitivity of mND to *k*.

- Supplementary Table 3. Runtimes

- Supplementary Table 4. Reported in a separate file.

**Supplementary References**

**Supplementary Methods**

**Choice of k evaluating the distribution of initial scores in the gene networks identified by mND**

The parameter *k* identifies the maximum number of neighbours that are considered in the calculation of mND score (see main text, Equations (3-4)). We have shown that considering at most 3 neighbours is a reasonable trade-off (Figure 2 and Figure S5) and that varying *k* of one unit had only minor effects on mND scores, which are highly correlated (Supplementary Figure S2 and Table S2). An opportunity to further optimize the value of *k* in front of a new dataset relies in selecting a value that yields connected networks enriched in initial scores. To this aim, we adapted the Ω function at the basis of network resampling method (Bersanelli et al., 2016; Mosca et al 2017). Indeed, we apply such function (designated here as ${}_{0}$) to the original scores $\mathbf{X}\left( R_{kn},l \right)$ in layer *l* associated with the top $n$ genes $(R_{kn})$ ranked by mND using a particular *k* value:

$${}_{0}\left( \mathbf{X}\left( R_{kn},l \right),\mathbf{A}\left( R_{kn},l \right) \right)={\mathbf{X}\left( R_{kn},l \right)}^{T}\mathbf{A}\left( R_{kn} \right) \mathbf{X}\left( R_{kn},l \right)=\omega_{knl}$$

where $\mathbf{A}\left( R_{kn} \right)$ is the adjacency matrix relative to $R_{kn}$. The resulting value $\omega_{knl}$ increases as the initial scores of the top ranking connected genes increases. We define the global trend of $\omega_{knl}$ over all layers among the top $i\in\{1, 2,\ldots,n\}$ ranking genes at varying *k*, summing the $\omega_{knl}$ values of each layer, normalized by the maximum value observed in such layer using different *k*:

$$\omega_{ki}^{'}=\sum_{l=1}^{L} \frac{\omega_{kil}}{\max_{ki} \left( \omega_{kil} \right)}$$

The non-decreasing trend of $\omega_{ki}^{'}$ varies in the interval $\left[ 0,L \right]$ and highlights the effect of *k* on the connectivity of top *n* ranking genes found by mND and the presence of high initial scores in such gene networks (e.g. Supplementary Figure S3).

**Supplementary Figures**


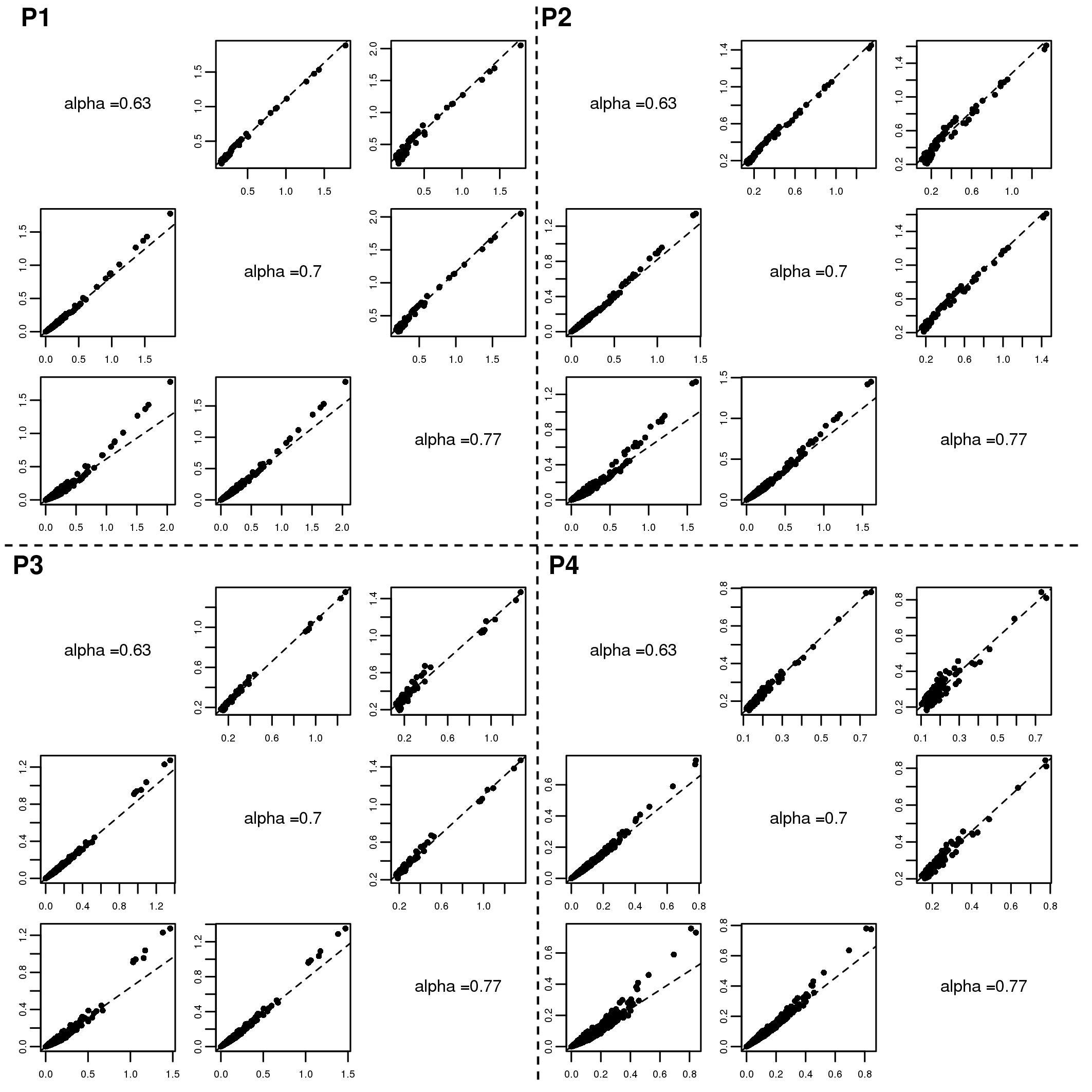


Supplementary Figure S1. Sensitivity of mND to *α* parameter. Correlation of mND scores at varying *α*, reported for all genes (below diagonal) and top 100 genes only (above diagonal) in four examples (P1-P4) of the analysis described in section 2.4 of main text. See also Supplementary Table S2.


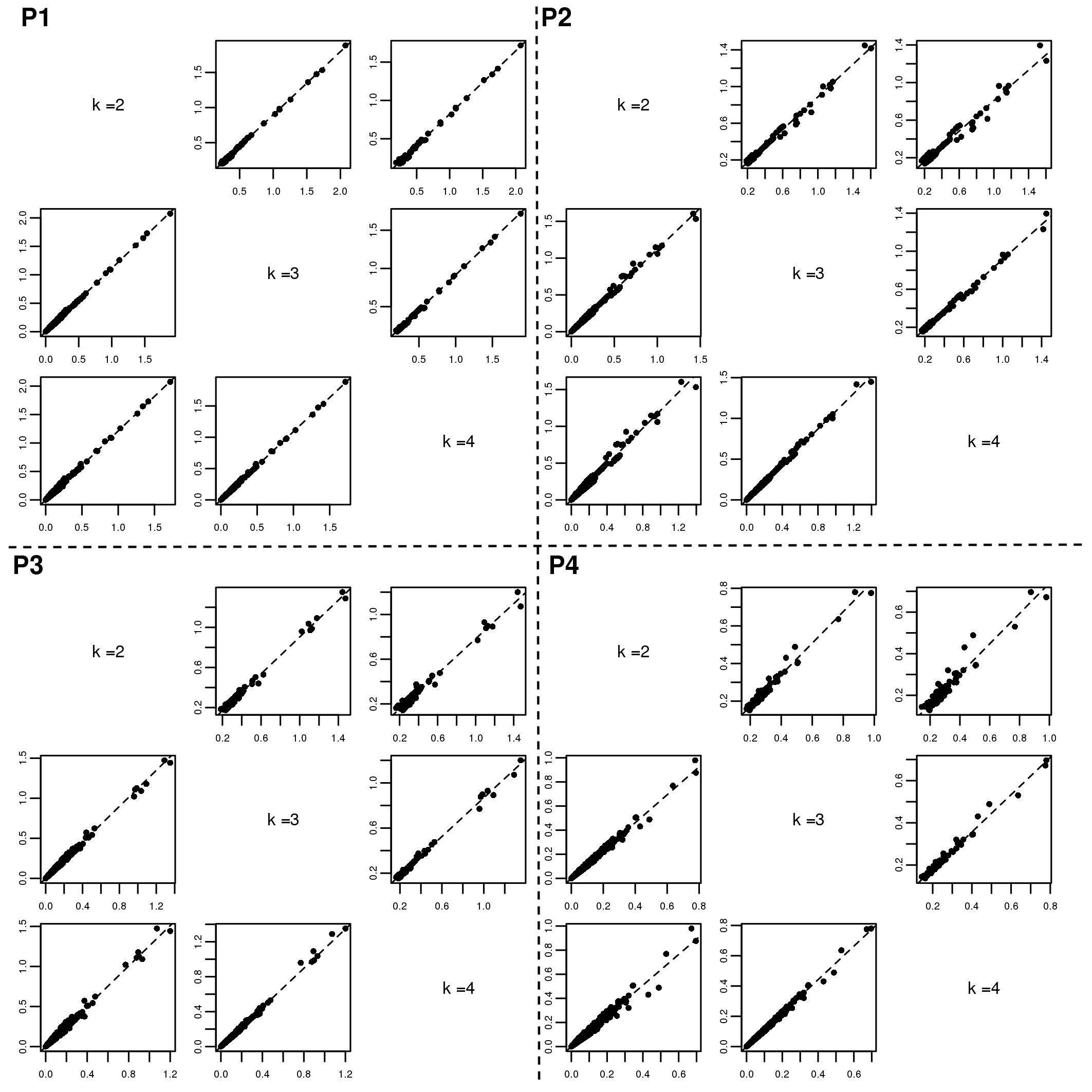


Supplementary Figure S2. Sensitivity of mND to the value of *k*. Correlation of mND scores at varying *k,* reported for all genes (below diagonal) and top 100 genes only (above diagonal) in four examples (P1-P4) of the analysis described in section 2.4 of main text. See also Supplementary Table S3.

$n$


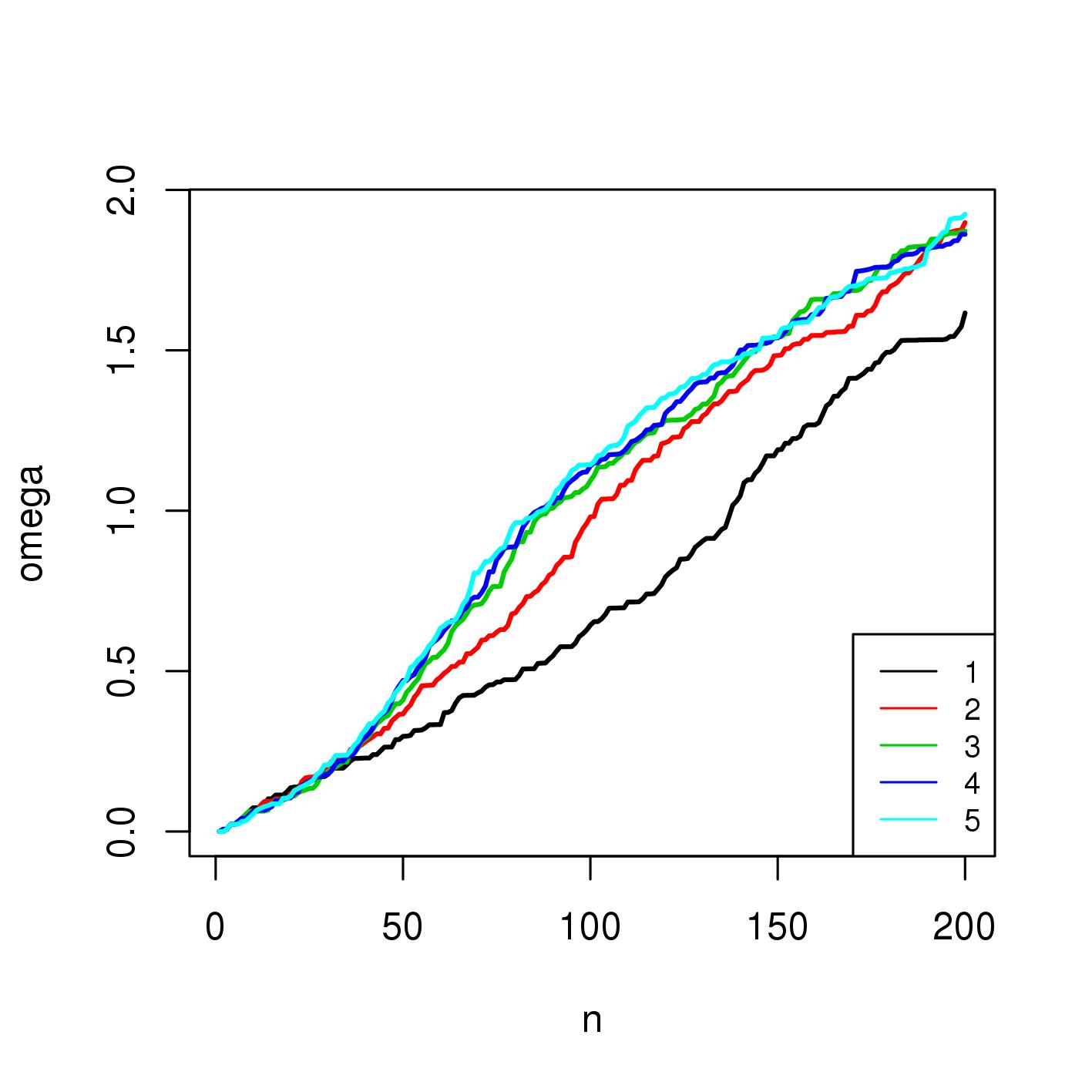

$$\omega_{\mathrm{ki}}^{'}$$

Supplementary Figure S3. Effect of varying *k* on the enrichment of the top gene networks in high initial scores. Trend of $\boldsymbol{\omega}_{\boldsymbol{ki}}^{\mathbf{'}}$ values (see Supplementary Methods) among the top 200 genes found by mND at varying values of *k* in the analysis of mutations and gene expression changes in BC.


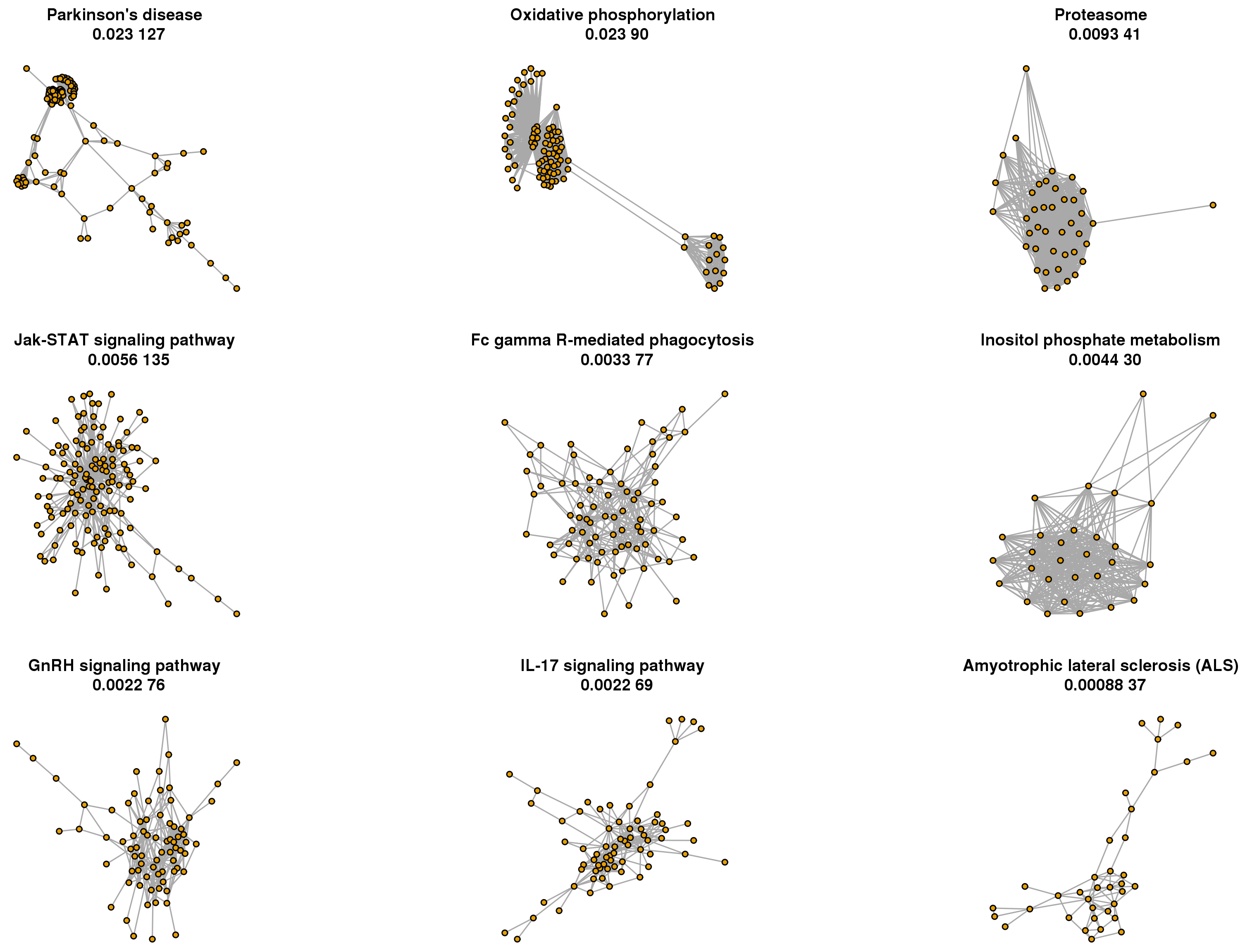


Supplementary Figure S4. Gene modules. Largest connected component of biological pathways from KEGG database (Kanehisa et al., 2017) in GH interactome. The two quantities below pathway name are modularity (as defined in (Clauset et al., 2004) and implemented in R function “modularity”) and size (number of genes).


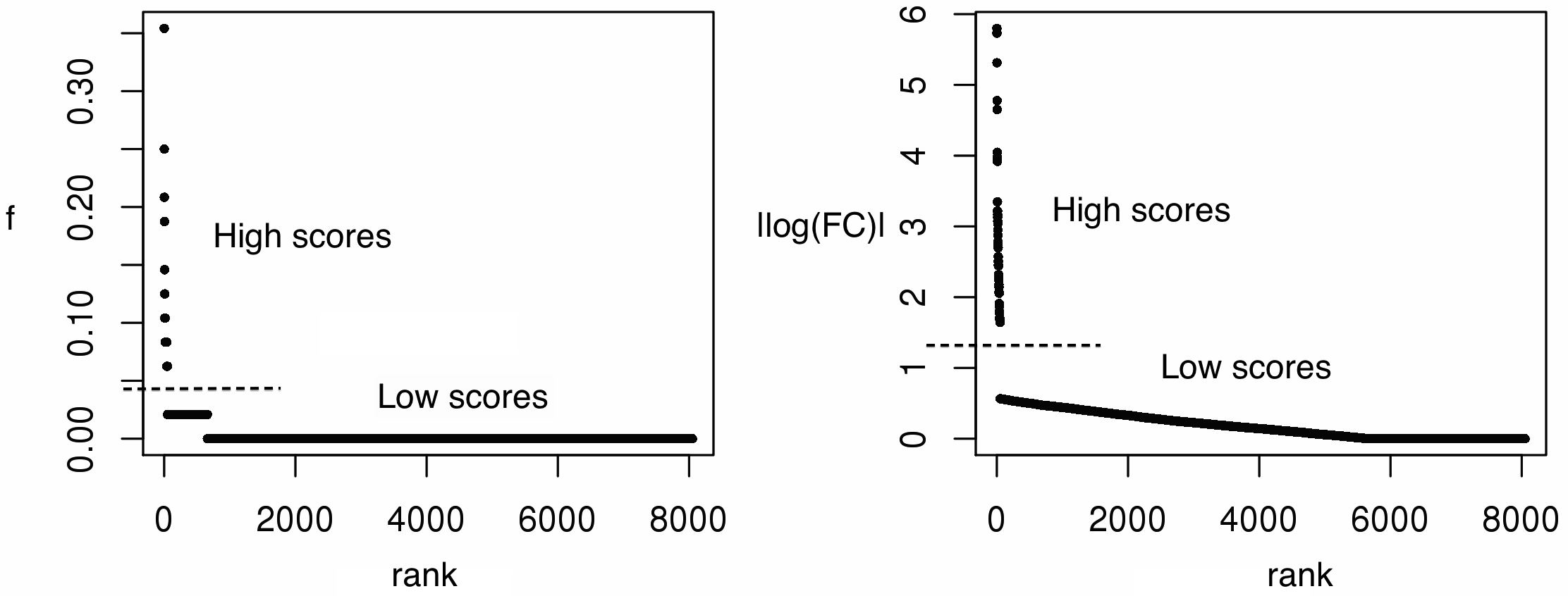
 Supplementary Figure S5. Initial gene scores assigned to gene modules. Two types of high and low scores that were randomly assigned to gene modules, derived from gene mutation frequency across subjects (left) and absolute fold changes between matched tumor-normal samples (right) from TCGA breast cancer data (see main text).


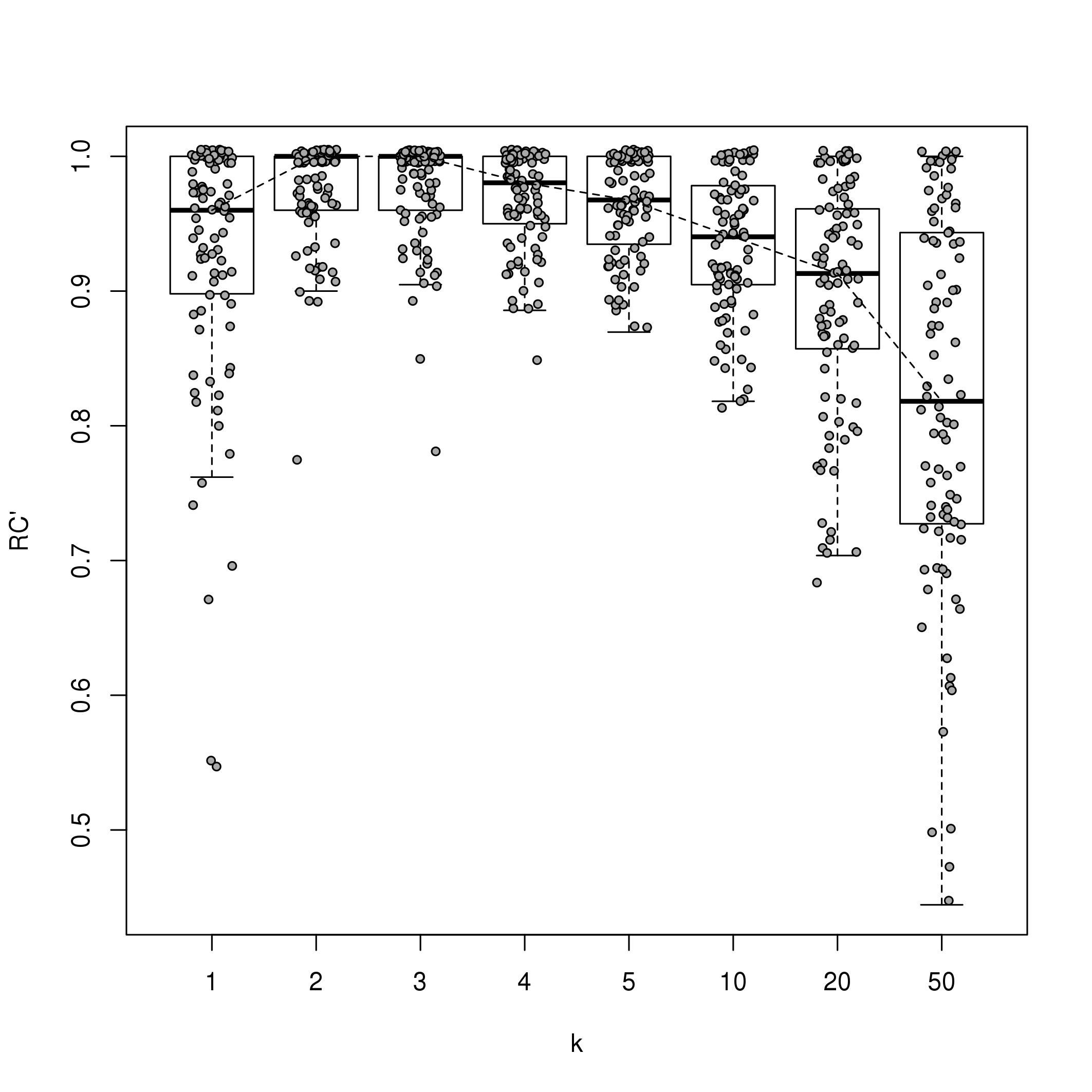


Supplementary Figure S6. Recall values in the analysis of 3 layers. Recall values normalized by the highest recall found for each input configuration at varying number of neighbors (*k*). This analysis was carried out like described in section 2.3 of the main text, but using 3 layers of mutation frequencies, like in a hypothetical analysis of three cancer subtypes or a hypothetical pan-cancer analysis.


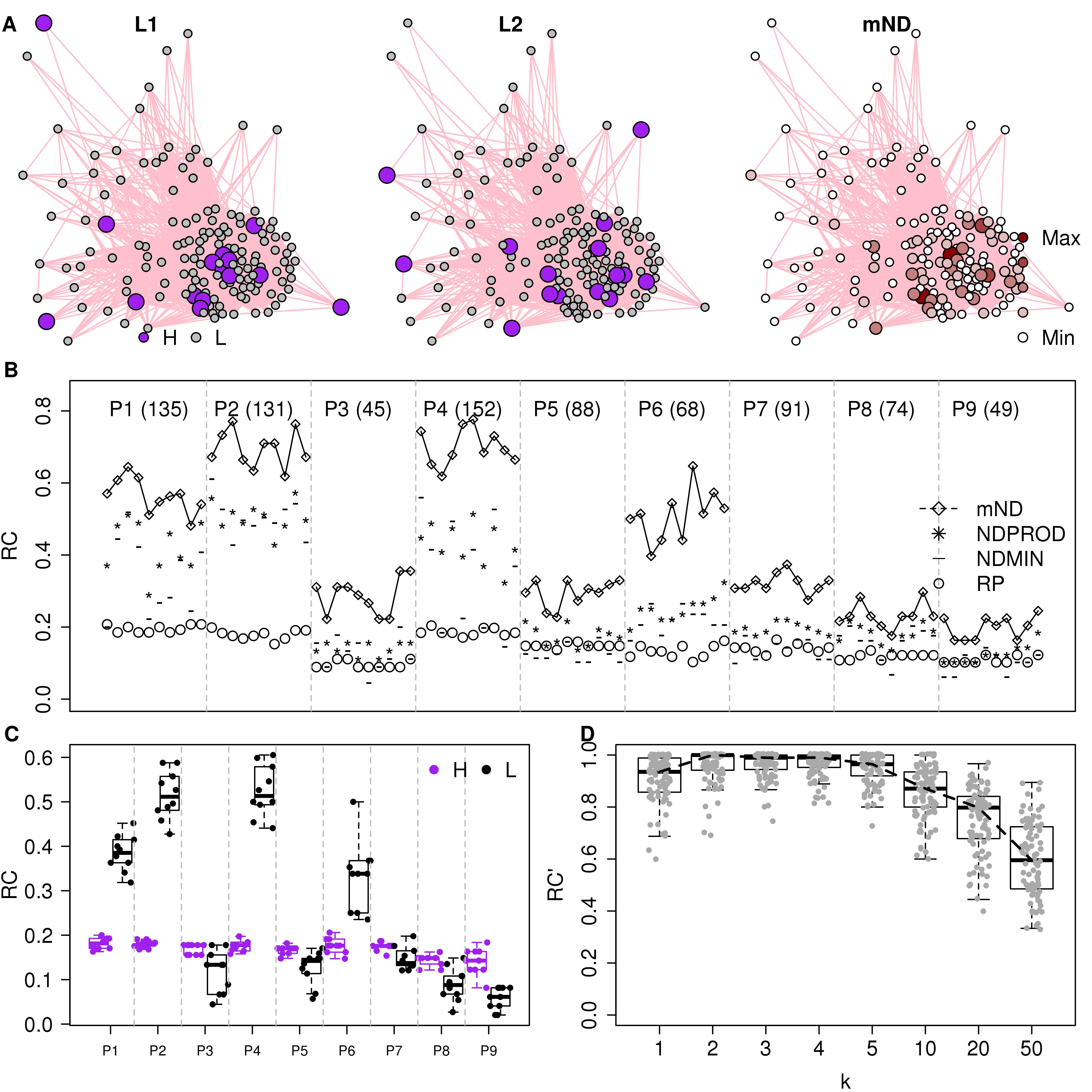


Supplementary Figure S7. Performance in ranking high scoring genes in network proximity in STRING. (A) Example of a gene module with its high scoring genes (H, purple) in each of the two layers and the resulting mND score; only genes belonging to the module and links occurring among such genes are reported. (B) Recall values for 10 signal permutations for each of the 9 modules (P1, P2, …, P9), using mND score and other methods; the number between parentheses after module id is module size. (C) Recall values, shown separately for high scoring genes and other genes in each module. (D) Recall values normalized by the highest recall found for each input configuration at varying number of neighbors (*k*). (A-D) These results were obtained carrying out the same analysis described in section 2.3 of the main text, but using interactome STRING.

**
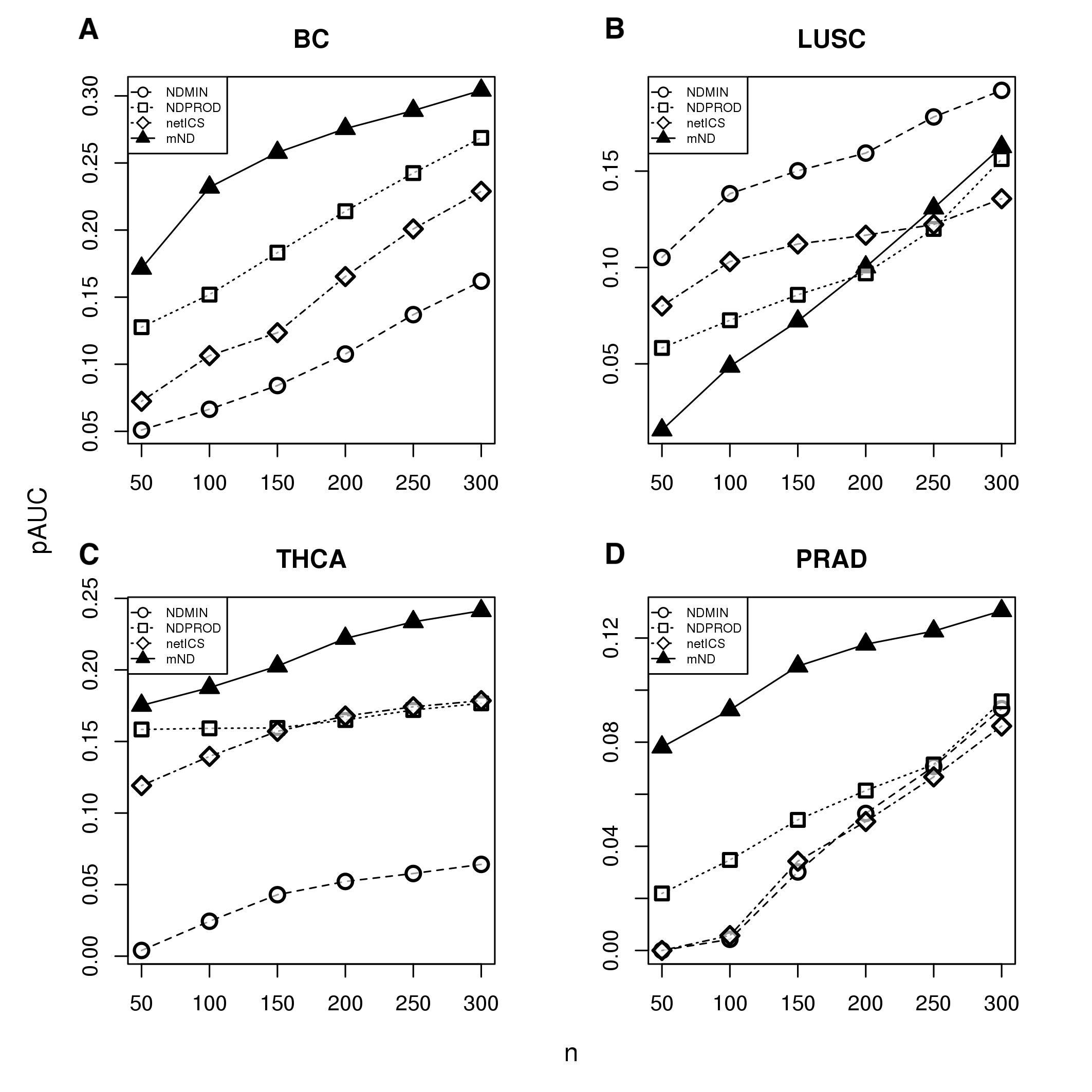
**

**Supplementary Figure S8**. **Performance in recovering mutated genes associated with cancer.** Partial AUC (pAUC) at varying number of top false positive ranking genes (n) in integration of mutation profiles of subjects. The reference gene set was composed of mutated genes. (A-D) These results were generated using interactome WU.

**
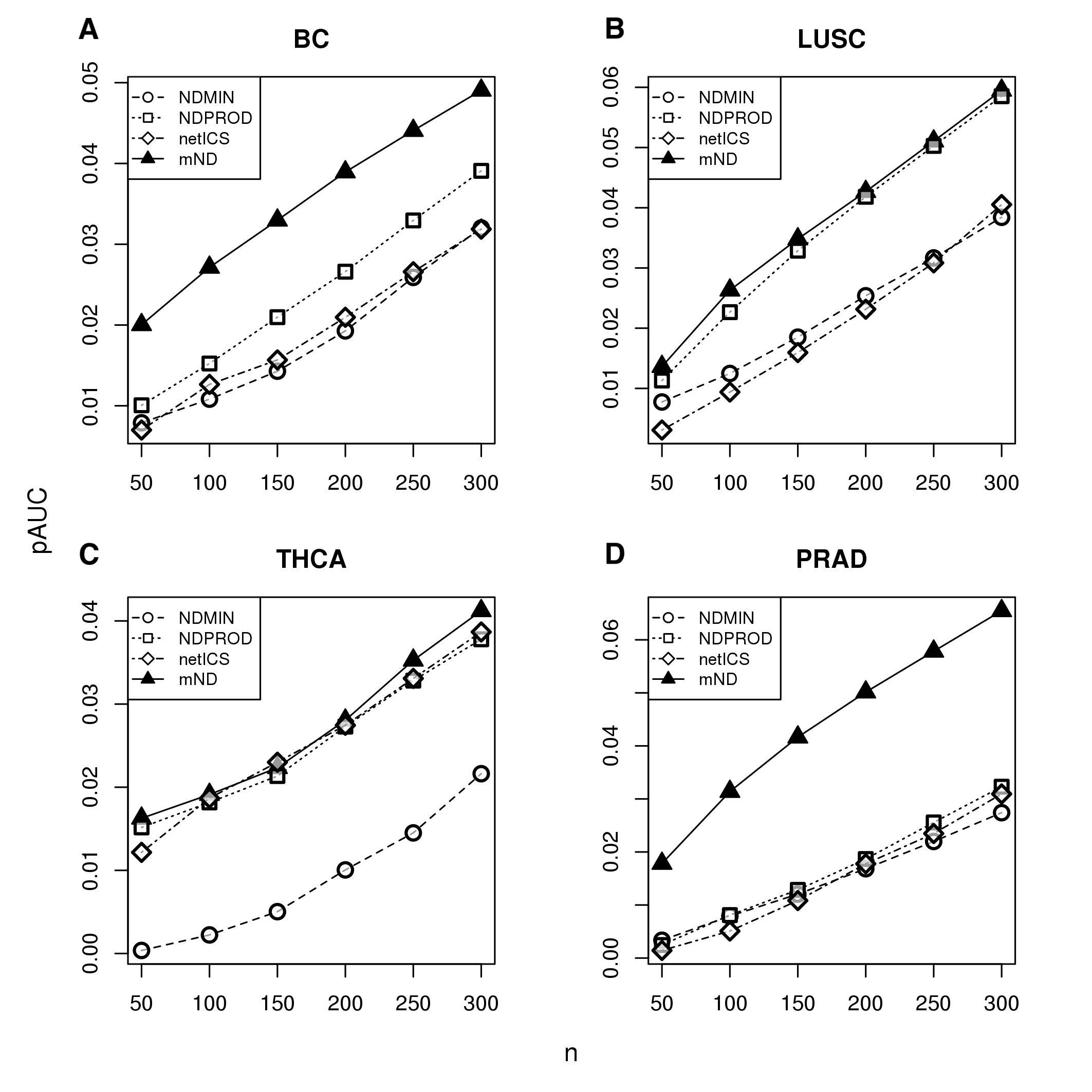
**

**Supplementary Figure S9**. **Performance in recovering known cancer genes.** Partial AUC (pAUC) at varying number of top false positive ranking genes (n) in integration of mutation profiles of subjects. The reference gene set was composed of both mutated genes and differentially expressed genes. (A-D) These results were generated using interactome WU.


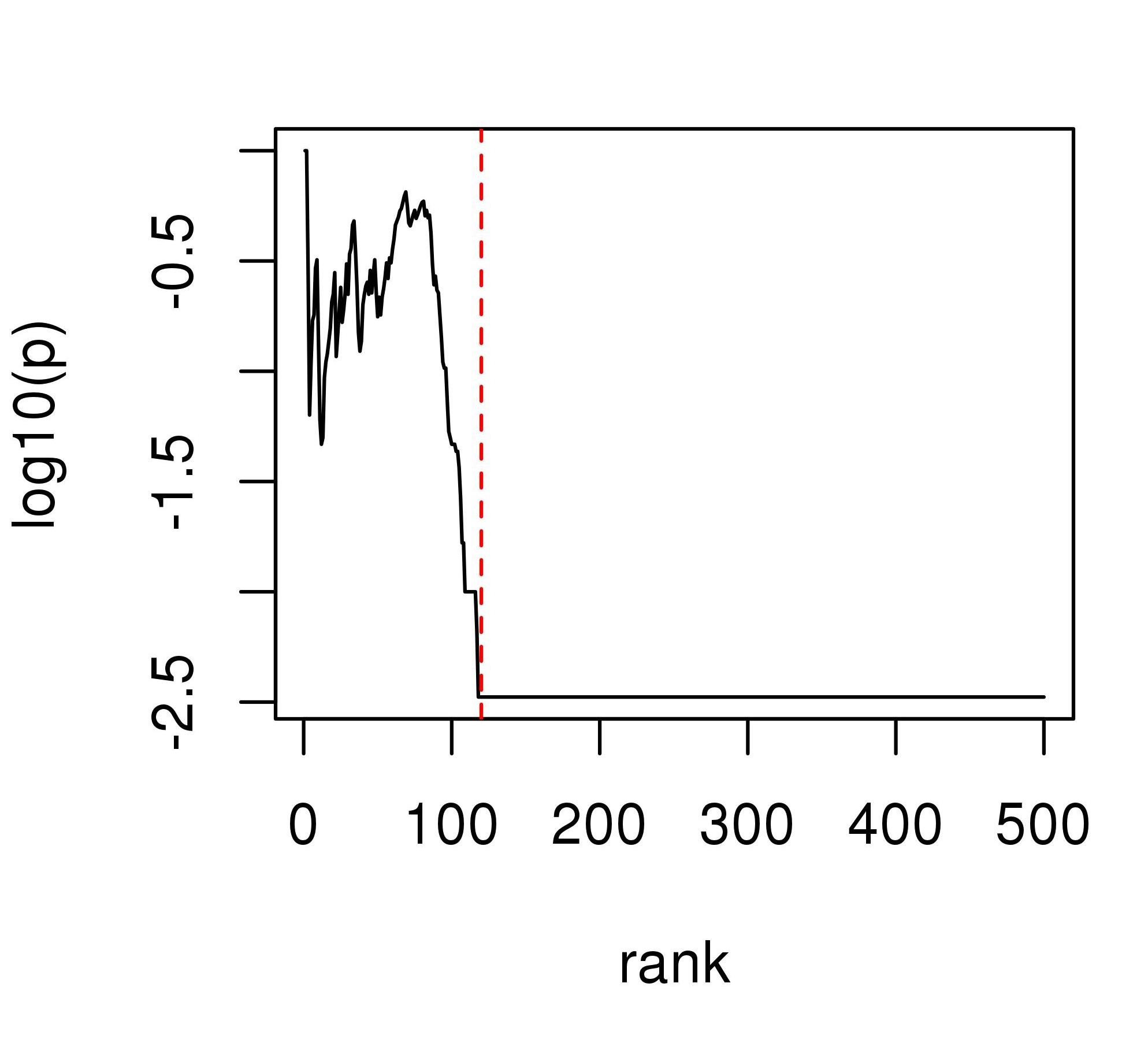


Supplementary Figure S10. Network resampling on breast cancer data from TCGA. Logarithm of *p*-value (y-axis) calculated for each rank of a gene list (x-axis) ordered by decreasing values of mNDp.


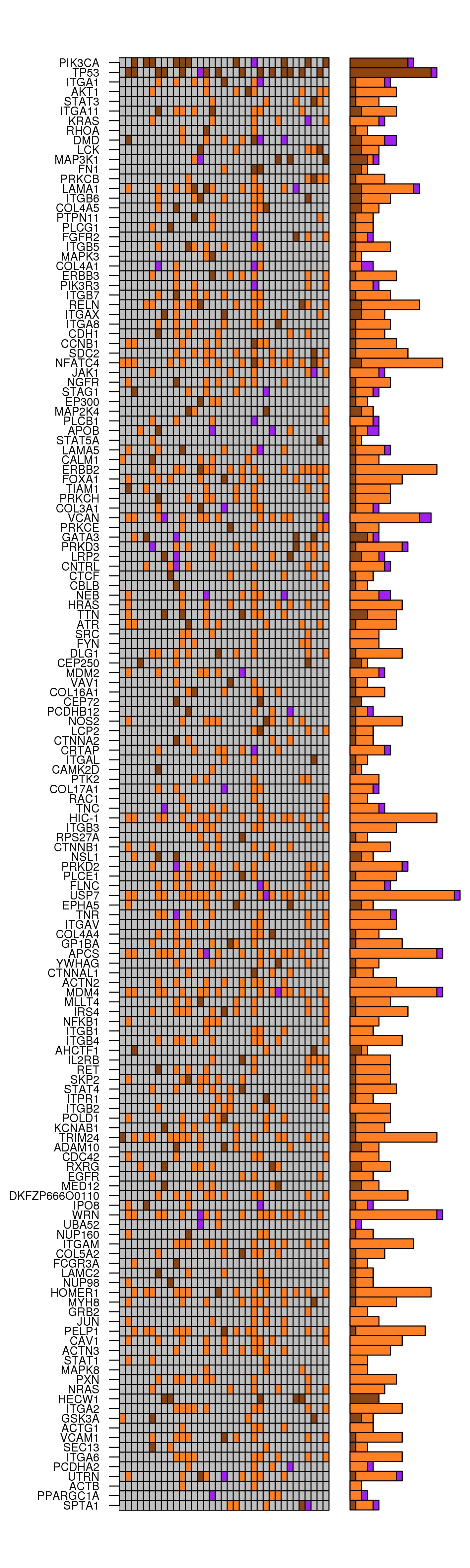


Supplementary Figure S11. Classification of genes across layers in the integration of mutation profiles of subjects. Top 150 ranked genes by mND; brown: isolated; orange: linker; purple: module; grey: not significant. Barplots reflect the occurrence of the different labels – isolated, linker, module – a gene may assume across layers. These results were generated using interactome WU and BC data from TCGA.

**Supplementary Tables**

| $\alpha$ | 0.63 | 0.7 | 0.77 |
| --- | --- | --- | --- |
| $\left\langle\% \right\rangle$ | 6.0 | 0 | 7.5 |

**Supplementary Table S1**. **Sensitivity of mND to** $\boldsymbol{\alpha}$. Average percentage ($\left\langle\% \right\rangle$) of genes that change within the top 100 ranked by mND in 90 runs (see main text subsection 2.4) varying $\alpha$ by ± 10%.

| *k* | 2 | 3 | 4 |
| --- | --- | --- | --- |
| $\left\langle\% \right\rangle$ | 5.8 | 0 | 4 |

**Supplementary Table S2**. **Sensitivity of mND to *k***. Average percentage ($\left\langle\% \right\rangle$) of genes that change within the top 100 ranked by mND in 90 runs (see main text subsection 2.4) varying *k* of 1 unit.

|  | **#P** | **Cores** | ***L*** | **Interactome** | **#V** | **#E** | **ND**  **[hh:mm:ss]** | **mND**  **[hh:mm:ss]** | **Total**  **[hh:mm:ss]** |
| --- | --- | --- | --- | --- | --- | --- | --- | --- | --- |
| **BC – T1** | 1000 | 4 | 2 | WU | 6 016 | 128 150 | 0:33:39 | 0:06:11 | 0:39:50 |
| **LUSC – T1** | 1000 | 4 | 2 | WU | 6 016 | 128 150 | 0:33:49 | 0:06:18 | 0:40:07 |
| **THCA – T1** | 1000 | 4 | 2 | WU | 6 016 | 128 150 | 0:19:57 | 0:06:18 | 0:26:15 |
| **PRAD – T1** | 1000 | 4 | 2 | WU | 6 016 | 128 150 | 0:32:53 | 0:05:56 | 0:38:49 |
| **BC – T1** | 1000 | 4 | 2 | STRING | 11 796 | 309 850 | 1:35:05 | 0:13:35 | 1:48:40 |
| **BC – T2** | 1000 | 4 | 35 | WU | 6 016 | 128 150 | 2:27:34 | 1:36:31 | 4:04:05 |
| **LUSC – T2** | 1000 | 4 | 23 | WU | 6 016 | 128 150 | 2:45:38 | 1:02:34 | 3:48:12 |
| **THCA – T2** | 1000 | 4 | 17 | WU | 6 016 | 128 150 | 1:45:34 | 0:45:42 | 2:31:16 |
| **PRAD – T2** | 1000 | 4 | 27 | WU | 6 016 | 128 150 | 1:48:30 | 1:13:49 | 3:02:19 |

Supplementary Table S3. Runtimes. Total run times are split in “ND”, the time required up to and including network diffusion, and “mND”, the following part of the pipeline. “-T1” refers to the analysis of mutations and expression change, “-T2” refers to the analysis of mutation profiles of multiple patients; #P: number of permutations; *L*: number of layers; #V: number of genes; #E: number of interactions.

**Supplementary References**

Bersanelli, M. et al*.* (2016) Network diffusion-based analysis of high-throughput data for the detection of differentially enriched modules, *Sci. Rep.*, 6, 34841.

Clauset, A et al. (2004) Finding community structure in very large networks, *Phyisical Review E*, 70, 066111.

Kanehisa, M. et al. (2017) KEGG: new perspectives on genomes, pathways, diseases and drugs. *Nucleic Acids Research*, 45, 353-361.

Mosca, E. *et al.* (2017) Network Diffusion-Based Prioritization of Autism Risk Genes Identifies Significantly Connected Gene Modules. *Front. Genet*., 8:129.
